# Evaluating Latent Tuberculosis Infection Test Performance Using Latent Class Analysis in a TB and HIV Endemic Setting

**DOI:** 10.3390/ijerph16162912

**Published:** 2019-08-14

**Authors:** Shahieda Adams, Rodney Ehrlich, Roslynn Baatjies, Nandini Dendukuri, Zhuoyu Wang, Keertan Dheda

**Affiliations:** 1 School of Public Health and Family Medicine, Division of Occupational Medicine, University of Cape Town, Observatory 7925, South Africa; 2 Department of Environmental and Occupational Studies, Faculty of Applied Sciences, Cape Peninsula University of Technology, Cape Town 8000, South Africa; 3 Division of Clinical Epidemiology, McGill University Health Centre—Research Institute, Montreal, QC H4A 3J1, Canada; 4 Centre for Lung Infection and Immunity, Division of Pulmonology, Department of Medicine and UCT Lung Institute & South African MRC/UCT Centre for the Study of Antimicrobial Resistance, University of Cape Town, Observatory, Cape Town 7925, South Africa; 5 Faculty of Infectious and Tropical Diseases, Department of Immunology and Infection, London School of Hygiene and Tropical Medicine, London WC1E 7HT, UK

**Keywords:** latent class analysis, latent tuberculosis infection, health care worker

## Abstract

Background: Given the lack of a gold standard for latent tuberculosis infection (LTBI) and paucity of performance data from endemic settings, we compared test performance of the tuberculin skin test (TST) and two interferon-gamma-release assays (IGRAs) among health-care workers (HCWs) using latent class analysis. The study was conducted in Cape Town, South Africa, a tuberculosis and human immunodeficiency virus (HIV) endemic setting Methods: 505 HCWs were screened for LTBI using TST, QuantiFERON-gold-in-tube (QFT-GIT) and T-SPOT.TB. A latent class model utilizing prior information on test characteristics was used to estimate test performance. Results: LTBI prevalence (95% credible interval) was 81% (71–88%). TST (10 mm cut-point) had highest sensitivity (93% (90–96%)) but lowest specificity (57%, (43–71%)). QFT-GIT sensitivity was 80% (74–91%) and specificity 96% (94–98%), and for TSPOT.TB, 74% (67–84%) and 96% (89–99%) respectively. Positive predictive values were high for IGRAs (90%) and TST (99%). All tests displayed low negative predictive values (range 47–66%). A composite rule using both TST and QFT-GIT greatly improved negative predictive value to 90% (range 80–97%). Conclusion: In an endemic setting a positive TST or IGRA was highly predictive of LTBI, while a combination of TST and IGRA had high rule-out value. These data inform the utility of LTBI-related immunodiagnostic tests in TB and HIV endemic settings.

## 1. Introduction

The testing for and treatment of latent tuberculosis (TB) infection (LTBI) in targeted populations is an important strategy in TB elimination [1,2]. In South Africa targeted LTBI testing is aimed at children under the age of five years as infection is one of the criteria used in the diagnosis of TB. In adults, it is used to diagnose latent infection in immunosuppressed patients, specifically human immunodeficiency virus (HIV) positive individuals and silicotic gold miners who would benefit from isoniazid (INH) prophylaxis [3]. The benefit of LTBI treatment is well established in HIV positive individuals with a substantial risk reduction of 35% (risk ratio (RR) = 0.65, 95% confidence interval (CI) 0.51, 0.84) in progression to TB disease, and an even larger effect in those who test tuberculin skin test (TST) positive for LTBI 52% (RR = 0.48; 95% CI 0.29, 0.82) [2]. The World Health Organization (WHO) recommends that in low TB incidence countries, targeting of LTBI in at-risk populations such as prisoners, health-care workers (HCWs), immigrants from high TB burden countries, homeless people and people who use illicit drugs, should be considered [2].

HCWs in South Africa have been shown to have a very high prevalence of LTBI (range 48–84%) and of HIV infection ranging from 11–20% [4,5,6,7]. Together with their occupational exposure to TB, this places them at higher risk of active TB disease than the general population [8,9,10,11]. The WHO recommendation regarding LTBI testing does not currently extend to high-burden countries. Constraining factors include cost and sustainability, but also uncertainty about the utility of LTBI tests in these settings [2].

The absence of a gold standard for LTBI diagnosis has created uncertainty about test peformance [12]. Whilst TST remains the most widely used, test performance is suboptimal in Bacille Calmette-Guérin (BCG) vaccinated populations as cell-mediated immunity to tuberculin antigens may reflect exposure to *Mycobacterium bovis* bacillus found in the BCG vaccine. Cell-mediated immunity to tuberculin antigens may also reflect exposure to similar antigens from environmental mycobacteria or a previous tuberculosis infection that has been cleared (through immunological mechanisms or treatment) [12].

Two T-cell-based assays have been developed for diagnosing LTBI and have been commercially available. The one test is an enzyme-linked immunosorbent assay (ELISA), the QuantiFERON-TB Gold-in–tube (QFT-GIT) (Cellestis Ltd., Carnegie, Australia) which measures the production of antigen-specific interferon-gamma (IFN-γ) by circulating T-cells in whole blood. The other is the T-SPOT.TB (Oxford Immunotec, Oxford, UK) which uses an Elispot technique to measure peripheral blood mononuclear cells (PMBCs) that produce IFN-γ. Both assays are characterized by the use of more specific *M. Tuberculosis* antigens—the early secretory protein-6 (ESAT-6) and the culture filtrate protein-10 (CFP-10)—which are encoded by genes found in the region of difference (RD1) on the *M. tuberculosis* genome. These genes are not present in the genome of *M. bovis* BCG or certain non-tuberculous mycobacteria such as *M. avium*. However, interferon-gamma-release assays (IGRAs) come at a higher cost than TST, require good laboratory infrastructure, and are invasive, all of which may serve as barriers to their practical uptake in low-resource settings.

IGRAs are currently being used as alternatives to TST in existing screening programmes for LTBI in HCWs. IGRAs measure interferon gamma release by memory T-cells after stimulation with TB *mycobacterium*-specific antigens absent in BCG and most non-tuberculous mycobacteria (NTMs) and are, therefore, considered to be specific [12].

Limited research has been conducted on the utility of these screening test for HCWs in high TB incidence countries (defined as >100/100,000 cases per year). The absence of a “gold standard” test for LTBI limits the direct comparison between TST and IGRA test performance [13]. Latent class analysis (LCA), a Bayesian statistical method, allows the estimation of LTBI prevalence and diagnostic test sensitivity while recognizing that all available tests are imperfect [13].

Data from this study which evaluated incidence of LTBI in a subset of HCWs followed up from the same cohort have previously been published [9]. This part of the study compared the relative sensitivity, specificity and predictive value of the TST and both commercially available IGRAs for the diagnosis of LTBI in HCWs at baseline using an LCA model. To our knowledge, this is the first such comparative study incorporating LCA for all three tests in a high TB/HIV endemic setting. Only two similar studies have previously been performed, both based in low TB incidence settings, with only one focusing on HCWs [14,15,16].

## 2. Materials and Methods

### 2.1. Population and Test Outcomes

HCWs were drawn from seven healthcare facilities providing TB diagnostic and treatment services. Five facilities were located in the Cape Town suburb of Khayelitsha, a very high TB incidence area with a TB case notification rate of over 1, 600/100,000, 70% of whom are co-infected with HIV [17]. The study was approved by the Human Research Ethics Committee of the Faculty of Health Sciences at the University of Cape Town, South Africa (Reference Number: 417/2008; date of approval: 3 November 2008).

From an eligible study population of 764 HCWs, 505 were recruited to the study. All participants underwent administration of TST and venesection for QFT-GIT and T-SPOT.TB. TST was performed using 1 tuberculin unit (TU) dose of purified protein derivative (PPD) RT23 (Statens Serum Institut, Copenhagen, Denmark). Skin induration was read after 48–72 h using a ruler and ballpoint. An induration of at least 10 mm was considered positive. In the case of known HIV infection 5 mm or more was considered positive.

Blood samples for the IGRA assays were drawn concurrently or within three days of administering the TST to eliminate potential boosting, i.e., the generation of a false positive response to the recently administered tuberculin [18,19]. Administration and interpretation of IGRA test results were done in accordance with manufacturer’s instructions [9].

In view of the high TB incidence in this province, participants were also screened for current TB disease by way of a symptom screening questionnaire and chest radiograph. Those with a suspect radiograph or positive symptom screen were then referred for sputum microscopy and culture to confirm TB diagnosis. Statistical analyses were performed using Stata version 11 (Stata Corp, College Station, TX, USA). Outcomes included agreement between the tests using the kappa statistic (κ).

### 2.2. Latent Class Analysis

LCA is based on the premise that the results of various imperfect tests for a condition are influenced by a common underlying latent variable, which represents the true status [12,19]. We also used the model to calculate the positive and negative predictive values of each individual test and a combination of two tests, specifically a composite rule under which a HCW who is positive on either TST or QFT-GIT is classified as LTBI positive, and if negative on both tests classified as LTBI negative.

In applying LCA, subjects with both determinate and indeterminate results were included as participants with indeterminate results in one test may still have relevance in the analysis on account of determinate results in the remaining two tests.

Similarities in technological properties and immunological mechanism underlying the two IGRA assays could potentially result in correlation of the errors in both tests, referred to as conditional dependence. Therefore, a latent class model that allowed for conditional dependence between QFT and TSPOT.TB both among LTBI positive and LTBI negative subjects was considered [20,21]. A fixed effects model allowing for conditional dependence between QFT-GIT and TSPOT.TB was used to fit the data [22].

When the number of diagnostic tests used in the study sample does not provide at least as many degrees of freedom as the number of unknown parameters to be estimated, the model is not identifiable. This was the case for our analysis as the number of degrees of freedom available was seven, but the number of unknown parameters was nine [LTBI prevalence, sensitivity and specificity of the three individual tests, and two covariance terms between the two IGRA tests (among LTBI positive and LTBI negative individuals)]. To obtain a meaningful solution to this analytic problem it is necessary to employ a Bayesian approach for inference and to provide prior information (i.e., information external to the observed data) on some of the unknown parameters [23].

In this instance, prior information is available on the sensitivity and specificity of the tests from the literature (Table 1) [24]. The latent class analysis updates this prior information using the newly observed data to provide posterior distributions for each unknown test characteristic. Results of the Bayesian analysis are reported as the median and 95% credible intervals (the Bayesian equivalent of a confidence interval) for each test performance characteristic. Another advantage of using the Bayesian approach is that one can use the posterior distributions to calculate the probability that the performance characteristic of one test is greater than that of another test.

Prior information on the accuracy of the three tests was elicited from Pai, Zwerling and Menzies using ranges of sensitivity and specificity of TST and QFT-GIT based on systematic reviews and meta-analyses of studies, carried out in high TB burden settings and BCG-vaccinated populations. (Table 1) [24]. Each of these ranges was expressed as a Beta probability distribution by matching the end points of the range to the 2.5% and 97.5% quantiles of the distribution. A non-informative Beta (1,1) prior distribution was used for the prevalence of LTBI, which is unknown in this study. In other words, this prior information places equal weight on all values between 0% and 100%. The prior 95% credible interval (CrI) of each parameter was set to be equivalent to the confidence interval resulting from the meta-analysis.

Non-informative prior distributions were used for the covariance terms over their possible ranges. In an attempt to use as little prior information as possible, we first fitted a model that incorporated prior information on specificities only. We then fitted a model that incorporated prior information on both specificities and sensitivities for comparison.

WinBUGS software was used to analyze the data [25]. Twenty thousand samples were drawn from the posterior distribution after discarding a burn-in of 1000 iterations. Convergence of the Monte Carlo Markov chain was assessed using the Gelman–Rubin statistic.

## 3. Results

Participants were predominantly female (74%) with a high prevalence of BCG vaccination (84%) and previous TB (13%). LTBI test positivity was 84% using TST, 65% using QFT-GIT and 60% using T-SPOT-TB (Table 2).

As a secondary objective, the prevalence of active TB in this population was also evaluated. Screening for current active TB demonstrated a high prevalence of chest radiograph abnormality (23%) (X-ray compatible with active or inactive TB) and a positive TB symptom screen (26%) (defined as yes to the presence of any TB symptoms). Those who were HIV positive (on testing or as reported) were 22 (11%).

Two participants indicated that they were currently receiving TB treatment, one of whom was HIV positive. Of the 103 participants referred for sputum investigation (those who were either symptom screen positive or had a chest radiograph in keeping with active TB), a further five tested positive for active disease. This translates into a prevalence of 5/503 or 1/1000 for active TB in this population. Of the five new cases detected, none tested positive on sputum microscopy and all five were culture positive. Three tested positive on symptom screen, three on chest radiograph, and three were HIV positive. One case did not test positive on either chest radiograph or symptom screen but was referred for sputum at her own request as she had felt unwell and had a colleague recently diagnosed with TB as a result of the study. This represented a deviation from the study protocol. All cases were TST positive, three were QFT and four were T-SPOT.TB positive.

Agreement analysis revealed poor agreement for test positivity when comparing TST to QFT-GIT (kappa = 0.28) and to TSPOT.TB (kappa = 0.25) (Table 3).

Using prior information on specificities only, LTBI prevalence was estimated at 81% (95% CrI 71–88%), with the sensitivity highest for TST (Table 4). The probability that the sensitivity of TST was higher than that of QFT was 0.99, and higher than that of TSPOT.TB, 1.0. Specificity on the other hand was significantly lower for TST than for the IGRAs (57% vs. 95–96%).

A model using prior information on the sensitivities as well as specificities of all three tests estimated the prevalence of LTBI to be 76% (95% CrI 70–83%). TST sensitivity was again higher than that of IGRAs (Table 5). In this model the probability that the sensitivity of TST was higher than that of QFT was 0.95 and of TSPOT.TB 0.88.

In the model that incorporated prior information on specificity only, the positive predictive value, i.e., the proportion of positive tests correctly identifying the presence of LTBI of all tests was high: QFT-GIT 98% TSPOT.TB 99% and TST 90%. However, the negative predictive value, i.e., the proportion of negative tests correctly identifying the absence of LTBI, of all the tests was relatively low (range 47–65%) (Table 4).

On a composite rule classifying a participant as LTBI positive if either TST or QFT-GIT was positive, the positive predictive value remained high at 90% (95% CrI 81–95%) when compared to individual tests. By contrast, the negative predictive value (i.e., of both tests negative) was substantially greater than those of the individual tests, at 90% (95% CrI 80–97%).

## 4. Discussion

The main findings of this study in a TB/HIV endemic setting are that (i) TST is highly sensitive for LTBI diagnosis and strongly predictive of the presence of LTBI; (ii) IGRAs have superior specificity for LTBI diagnosis, and (iii) a combination of TST and IGRA has high rule-out value for LTBI (strong negative predictive value for the absence of LTBI). Participants had a high prevalence of LTBI, active TB and history of previous TB treatment. These findings reflect the endemic nature of TB in the underlying population and by implication in the occupational setting.

The LTBI prevalence as measured by TST, QFT-GIT and TSPOT.TB was 84%, 65% and 60% respectively for the group. The prevalence of LTBI as measured by TST is markedly higher than from a recent study of South African HCWs in Johannesburg which reported a 57% prevalence conducted among nurses and medical students [7]. It is, however, similar to community based general population studies in the Western Cape, which reported an LTBI prevalence of 81–88% [26,27,28]. Wood et al., (2010) showed an increasing prevalence of latent TB infection with increasing age in a population of HIV negative individuals aged 5–40 years drawn from high TB prevalence areas, with 88% of adults 31–35 years of age testing positive on TST. The prevalence of LTBI among.

HCWs in this study is therefore not indicative of greater TB infection risk than is found in community participants in the Western Cape. A more recent review of LTBI in HCWs in low- and middle-income countries by Apriani et al. reported the prevalence of LTBI in HCWs as ranging from 8–98% (mean 49%) based on TST, although studies were characterized by a high degree of heterogeneity [16]. Whilst sensitization to environmental mycobacteria may play a role in LTBI prevalence as measured by TST, this is not considered a clinically important cause of false-positive TST results, except in populations with a high prevalence of NTM sensitization and a very low prevalence of TB infection [29]. It has also been shown that TST reactions greater than 10 mm are unlikely to be caused by NTM. It is noted that the median size of the TST reaction in this group was 18 mm (interquartile range (IQR) 13–22) [30,31]. Data on sensitization to environmental mycobacteria are not available for South Africa.

The high prevalence of LTBI as reflected in TST positivity is unlikely to be confounded by high rates of active TB in this population as only 1% participants were found to have active TB. Furthermore, TST has previously been found to be highly sensitive for LTBI diagnosis and has been shown to have equivalent predictive value for incident active TB as IGRAs [32].

The lower LTBI prevalence estimated by using IGRAs is in keeping with studies among HCWs from low and intermediate incidence TB burden settings which have generally reported lower LTBI prevalence measured by IGRA, primarily using QFT-GIT, than for TST [33]. This has been ascribed to the IGRAs’ greater specificity and less confounding by BCG vaccination. Studies using IGRAs among HCWs from high TB incidence settings have produced varied results. Apriani et al. reported the prevalence of positive IGRA as ranging from 9% to 86% (*p*-value for heterogeneity = 0.01) in HCWs [16].

LTBI estimates using IGRAs in our study approximate those found in a community-based South African study involving healthy adults, which performed a head-to-head comparison between TST and IGRAs [26]. As with TST, LTBI prevalence as measured by IGRAs was similar in HCWs and the community, reaffirming the high background prevalence of TB infection in this population.

In the absence of a gold standard for LTBI, the use of latent class analysis allowed a more direct comparison of TST and IGRA test performance in this population. Both models showed a higher sensitivity for TST than IGRAs for LTBI diagnosis, although the probability was somewhat attenuated in the model that included prior information on both sensitivity and specificity. Furthermore, the sensitivity of TST (93%) in this study is higher than that shown in BCG-vaccinated populations (84%) of immunocompetent adults [13]. Whilst our study included immune-compromised individuals, the impact if any would be to decrease TST sensitivity, not enhance it as anergy in immunosuppressed individuals would result in false negative responses. As a first line screening test TST thus has a high probability of being more sensitive than either IGRA at detecting LTBI in this population.

The positive predictive value for IGRAs in this population (each 99%) compared to TST (90%) is slightly higher than that previously shown in a high prevalence (>50%) setting, i.e., QFT-GIT = 88% and TST = 73% [13]. This is most likely influenced by the exceptionally high prevalence of LTBI and near universal BCG vaccination in this population. However, the routine use of IGRAs in this setting is currently not advocated and given resource constraints and test performance of TST (higher sensitivity and slightly lower positive predictive value (PPV) than IGRA), this is likely to remain the first line test for LTBI.

Our analysis relied on prior information that IGRAs had significantly better specificity at ruling out LTBI than TST [24]. This relationship persisted after updating the prior information with the observed data. This is similar to findings from a recent review for BCG vaccinated populations where specificity for TST ranged from (76–82%) and BCG vaccination was shown to reduce TST specificity by as much as 21% [13]. In our study, an even lower estimate of specificity for TST 57% (95% CI; 43–71%) was found, suggesting that there may be a greater impact of BCG than elsewhere or an effect of exposure to other mycobacteria. IGRAs thus clearly show superior specificity to TST, making them superior as rule-out tests in this setting. Specificity of IGRAs appears to be unaffected by immune status (immune competent vs. immune compromised) [34].

The definition of optimal test performance in any given setting depends on the exact objective of testing, LTBI prevalence, and the costs, acceptability and sustainability of the proposed regimen. Therefore, there is no general rule for test optimality in practice. In the setting of an LTBI treatment programme based on test conversion offered to HCWs in a very high LTBI prevalence setting, high test accuracy at baseline is a priority to identify those in need of surveillance given the limited capacity to support such programmes. IGRAs have reasonably high sensitivity, high specificity, high PPV but low negative predictive value (NPV) in this setting. IGRA test results have also shown great variability when their use has been assessed in serial screening programmes, as compared to TST. This complicates baseline test interpretation [35,36,37,38]. There is therefore an argument for using a combined test, such as TST and QFT-GIT, at baseline. This takes advantage of TST’s high sensitivity and the greatly increased NPV from using a “both TST and IGRA negative” definition to produce a more accurate estimation of likely LTBI diagnosis. This will inform a more targeted approach to surveillance of those truly at risk and may result in cost-savings and less chance of unnecessary treatment for LTBI.

A limitation of the study is that of possible selection bias due to voluntary participation. Clinical staff (nurses and physicians) was underrepresented, probably as a result of high workloads and limited time to participate in the study during work hours, resulting in a greater proportion of participants being support and administrative staff. The LTBI prevalence may, therefore, be more reflective of high background community rates of infection in additional to any occupational effect than would have been the case with a higher proportion of physicians. However, many nurses come from high TB burden communities.

The study setting was one of the highest TB incidence areas in the country and findings may not be generalizable to all HCWs in South African or occupational settings where the community incidence of TB may be lower and occupational exposure less than is the case in this study. Furthermore, the use of cut-points for test positivity was based on the manufacturer’s instructions and software provided for assay analysis at the time that the study was conducted (≥6 spots or more for TSPOT.TB and ≥0.35 IU/mL for QFT-GIT). Current evidence suggests that further revision of cut points may be required which may be considerably different to those currently in use [37,38].

Another limitation of this study is the lack of sufficient sample size to evaluate test performance separately in immune competent and immune compromised individuals. Doan et al., in an extensive review evaluating LTBI test performance utilizing latent class analysis and Bayesian modelling, have shown that both TST and QFT-GIT have suboptimal sensitivity in immune compromised individuals [13]. Given the high HIV prevalence in South African HCWs (at least 10%) and the fact that such HIV positive HCWs face a six-fold higher risk of contracting TB than uninfected individuals, test performance among HIV positive health workers needs further investigation [39]. Recent work suggests that T-SPOT may be less susceptible to false negative status among anergic health workers with LTBI, but this needs to be confirmed [40].

## 5. Conclusions

In conclusion, our findings indicate that in populations with high TB incidence, high LTBI prevalence and near universal BCG vaccination, TST has a high probability of being more sensitive than IGRAs at detecting LTBI but lacks specificity. The high NPV of both TST and IGRA negative in combination may suggest a role for such combined testing to limit the unnecessary administration of LTBI treatment in resource limited settings where TB is endemic. The many factors other than test performance which determine the effectiveness of a programme of LTBI testing in HCWs in a high TB incidence setting need further research. These factors include, inter alia, prevalence of LTBI, conversion rates, risk of re-infection, objectives of the screening programme, opportunity costs of testing and treatment, acceptability of testing and LTBI treatment to HCWs, and the availability of occupational health service resources [41,42]. Cost-benefit analysis is needed to define an optimal testing strategy under different assumptions.

## Figures and Tables

**Table 1:** Prior information on sensitivity and specificity of tuberculin skin-test and interferon-gamma-release assays (IGRA).

Test Characteristic	Prior Distribution Range (95% CrI)		
	TST	QFT-GIT	TSPOT.TB
Sensitivity	71–82	63–78	86–93
Specificity	46–73	94–98	86–100

CrI: credible interval. Pai, Zwerling and Menzies, 2008. [24]. TST: Tuberculin skin test; QFT-GIT: QuantiFERON-TB Gold-in–tube.

**Table 2:** Demographic characteristics and test results of participants (N = 505).

Participant Characteristics	Number (%) of Participants
Gender	
Male	134 (26%)
Female	371 (74%)
Age	
<30 years	126 (25%)
31–40 years	136 (27%)
41–50 years	134 (27%)
>50 years	109 (22%)
History BCG vaccination	
Yes	423 (84%)
No	26 (5%)
Do not know	56 (11%)
Vaccination scar	398 (79%)
HIV positive	
Symptom screen positive	22 (11%)
Chest radiograph	131 (26%)
- Normal/other	381 (77%)
- Inactive TB	78 (16%)
- Suspect active TB	37 (7%)
Ever treated for TB	65 (13%)
Currently on TB treatment	2 (0.4%) 5 (1%)
Newly diagnosed with TB	
* Median TST reading (IQR)	18 mm (13–22)
TST Positive (N = 484)	405 (84%) (95% CI 80–87%)
QFT-GIT positive (N = 496)	324 (65%) (95% CI 61–70%)
TSPOT-TB positive (N = 465)	277 (60%) (95% CI 55–64%)

* For TST reading data presented as median and interquartile range (IQR). CI: confidence interval, N = number tested. BCG: Bacille Calmette-Guérin; TST: Tuberculin skin test; QFT-GIT: QuantiFERON-TB Gold-in–tube.

**Table 3:** Agreement and discordance between TST and IGRA assays.

	TST Versus IGRAS	
	QFT-GIT (N = 482)	TSPOT.TB (N = 450)
Positive TST and positive IGRA assay	293	249
Negative TST and negative IGRA assay	53	55
Positive TST and Negative IGRA assay	112	126
Negative TST and positive IGRA assay	24	20
Agreement (%)	71.8	67.6
Kappa (95% CI)	0.28 (0.20–0.36)	0.25 (0.18–0.33)

IGRAs: interferon-gamma-release assays; TST: Tuberculin skin test; QFT-GIT: QuantiFERON-TB Gold-in–tube; CI: confidence interval. TST ≥ 10 mm or ≥ 5 mm if HIV positive used to denote a positive TST test response.

**Table 4:** Performance of diagnostic tests for latent tuberculosis infection in healthcare workers using a latent class model using more informative prior information on specificities only (N = 472).

LTBI Test	Sensitivity % (95% CrI)	Specificity % (95% CrI)	PPV% (95% CrI)	NPV% (95% CrI)
TST	93 (90–96)	57 (43–71)	90 (80–95)	65 (50–79)
QFT-GIT	80 (73–90)	96 (94–98)	99 (98–99)	54 (35–79)
T-SPOT.TB	74 (67–83)	95 (89–99)	98 (96–100)	47 (30–69)
TST or QFT-GIT *	99 (98–99)	56 (42–69)	90 (81–95)	90 (80–97)

LTBI: latent tuberculosis infection; CrI: credible interval; TST: tuberculin skin test; QFT-GIT: QuantiFERON-Gold-in tube; PPV: positive predictive value; NPV: negative predictive value. Covariance terms were incorporated into the model to account for conditional dependence between QFT-GIT and TSPOT.TB. Covariance among truly positive subjects was 0.09 (95% CrI 0.09–0.12) and covariance among truly negative subjects was 0.01 (95% CrI −0.001–0.04). * This used a composite rule which defined LTBI as a either a positive TST or positive QFT-GIT.

**Table 5:** Performance of diagnostic tests for latent tuberculosis infection in healthcare workers using a latent class model using more informative prior information on specificities and sensitivities (N = 472).

LTBI Test	Sensitivity % (95% CrI)	Specificity % (95% CrI)	PPV% (95% CrI)	NPV% (95% CrI)
TST	86 (83–89)	49 (40–59)	84 (78–90)	53 (43–62)
QFT-GIT	82 (78–86)	96 (94–98)	98 (97–99)	63 (50–73)
T-SPOT.TB	83 (79–87)	97 (93–99)	99 (97–100)	65 (51–76)
TST or QFT-GIT *	98 (97–99)	47 (38–57)	85 (80–91)	86 (78–91)

LTBI: latent tuberculosis infection; CrI: credible interval; TST: Tuberculin skin test; QFT-GIT: QuantiFERON-Gold-in tube; PPV: positive predictive value; NPV: negative predictive value. Covariance terms were incorporated into the model to account for conditional dependence between QFT-GIT and TSPOT.TB. Covariance among truly positive subjects was 0.05 (95% CrI 0.01–0.08) and covariance among truly negative subjects was 0.01 (95% CrI 0.00–0.03). * This used a composite rule which defined LTBI as a either a positive TST or positive QFT-GIT.

## References

[1] Dheda C., Barry C.E., Maartens G. (2016). Tuberculosis. Lancet.

[2] World Health Organization (2018). Latent Tuberculosis Infection: Updated and Consolidated Guidelines for Programmatic Management.

[3] Department of Health. South Africa National Tuberculosis Management Guidelines 2014. https://www.health-e.org.za/wp-content/uploads/2014/06/NTCP_Adult_TB-Guidelines-27.5.2014.pdf.

[4] Shisana O., Hall E., Chavea J., Schwabe C. (2004). HIV/AIDS prevalence South African health workers. S. Afr. Med. J..

[5] Kranzer K., Bekker L.G., van Schaik N., Thebus L., Dawson M., Caldwell J., Hausler H., Grant R., Wood R. (2010). Community health care workers in South Africa are at increased risk for tuberculosis. S. Afr. Med. J..

[6] Connelly D., Veriava Y., Roberts S., Tsotetsi J., Jordan A., DeSilva E., Rosen S., DeSilva B.M. (2007). Prevalence of HIV infection and median CD4 counts among health care workers in South Africa. S. Afr. Med. J..

[7] Van Rie A., McCarthy K., Scott L., Dow A., Venter W.D., Stevens W.S. (2013). Prevalence, risk factors and risk perception of tuberculosis infection among medical students and healthcare workers in Johannesburg, South Africa. S. Afr. Med. J..

[8] Grobler L., Mehtar S., Dheda K., Adams S., Babatunde S., van der Walt M., Osman M. (2016). The epidemiology of tuberculosis in health care workers in South Africa: A systematic review. BMC Health Serv. Res..

[9] Adams S., Ehrlich R., Baatjies R., van Zyl-Smit R.N., Said-Hartley Q., Dawson R., Dheda K. (2015). Incidence of occupational latent tuberculosis infection in South African healthcare workers. Eur. Respir. J..

[10] Claassens M.M., van Schalkwyk C., du Toit E., Roest E., Lombard C.J., Enarson D.A., Beyers N., Borgdorff M.W. (2013). Tuberculosis in healthcare workers and infection control measures at primary healthcare facilities in South Africa. PLoS ONE.

[11] O’Donnell M., Jarand J., Loveday M., Padayatchi N., Zelnick J., Werner L., Naidoo K., Master I., Osburn G., Kvasnovsky C. (2010). High incidence of hospital admissions with multidrug-resistant and extensively drug-resistant tuberculosis among South African health care workers. Ann. Intern. Med..

[12] Pai M., Denkinger C.M., Kik S.V., Rangaka M.X., Zwerling A., Oxlade O., Metcalfe J.Z., Cattamanchi A., Dowdy D.W., Dheda K. (2014). Gamma Interferon Release Assays for detection of *Mycobacterium tuberculosis* Infection. Clin. Microbiol. Rev..

[13] Doan T.N., Eisen D.P., Rose M.T., Slack A., Stearnes G., Mc Bryde E.S. (2017). Interferon-gamma release assays for the diagnosis of latent TB infection: A latent-class analysis. PLoS ONE.

[14] Girardi E., Angeletti C., Puro V., Sorrentino R., Magnavita N., Vincenti D., Carrara S., Butera O., Ciufoli A.M., Squarcione S. (2009). Estimating diagnostic accuracy of tests for latent tuberculosis infection without a gold standard among healthcare workers. Eurosurveillance.

[15] Stout J.E., Wu Y., Ho C.S., Pettit A.C., Feng P.-J., Katz D.J., Ghosh S., Venkatappa T., Luo R. (2018). Evaluating latent tuberculosis infection diagnostics using latent class analysis. Thorax.

[16] Apriani L., McAllister S., Sharples K., Alisjahbana B., Ruslami R., Hill P.C., Menzies D. (2019). Latent Tuberculosis infection in health care workers in low and middle-income countries: An updated systematic review. Eur. Respir. J..

[17] Garone D., Hilderbrand K., Boulle A.M., Coetzee D., Goemaere E., Van Cutsem G., Besada D. (2011). Review: Khayelitsha 2001–2011: 10 years of primary care HIV and TB programmes. S. Afr. J. HIV Med..

[18] Van Zyl-Smit R.N., Pai M., Peprah K., Meldau R., Kieck J., Juritz J., Badri M., Zumla A., Sechi L.A., Bateman E.D. (2009). Within-subject variability and boosting of T-cell interferon-gamma responses after tuberculin skin testing. Am. J. Respir. Crit. Care Med..

[19] Van Zyl-Smit R.N., Zwerling A., Dheda K., Pai M. (2009). Within-subject variability of interferon-g assay results for tuberculosis and boosting effect of tuberculin skin testing: A systematic review. PLoS ONE.

[20] Pai M., Dendukuri N., Wang L., Joshi R., Kalantri S., Rieder H.L. (2008). Improving the estimation of tuberculosis infection prevalence using T-cell based assay and mixture models. Int. J. Tuberc. Lung. Dis..

[21] Dendukuri N., Joseph L. (2001). Bayesian Approaches to Modeling the Conditional Dependence between Multiple Diagnostic Tests. Biometrics.

[22] Dendukuri N., Hadgu A., Wang L. (2009). Modeling conditional dependence between diagnostic tests: A multiple latent variable model. Stat. Med..

[23] Joseph L., Gyorkos T., Coupal L. (1995). Bayesian Estimation of Disease Prevalence and the parameters of Diagnostic Tests in the Absence of a Gold Standard. Am. J. Epidemiol..

[24] Pai M., Zwerling A., Menzies D. (2008). Systematic Review: T-cell-based Assays for the Diagnosis of Latent Tuberculosis Infection: An update. Ann. Intern. Med..

[25] Lunn D.J., Thomas A., Best N., Speigelhalter D. (2000). WinBUGS-a Bayesian modelling framework: Concepts, structure and extensibility. Stat. Comput..

[26] Mahomed H., Hughes E.J., Hawkridge T., Minnies D., Simon E., Little F., Hanekom W.A., Geiter L., Hussey G.D. (2006). Comparison of Mantoux skin test with three generations of a whole blood IFN-gamma assay for tuberculosis infection. Int. J. Tuberc. Lung Dis..

[27] Middelkoop K., Bekker L.G., Myer L., Dawson R., Wood R. (2008). Rates of tuberculosis transmission to children and adolescents in a community with a high prevalence of HIV infection among adults. Clin. Infect. Dis..

[28] Wood R., Liang H., Wu H., Middelkoop K., Oni T., Rangaka M., Wilkinson R., Bekker L., Lawn S.D. (2010). Changing prevalence of tuberculosis infection with increasing age in high-burden township in South Africa. Int. J. Tuberc. Lung Dis..

[29] Farhat M., Greenaway C., Pai M., Menzies D. (2006). False-positive tuberculin skin tests: What is the absolute effect of BCG and nontuberculous mycobacteria?. Int. J. Tuberc. Lung Dis..

[30] Bugiani M., Borracino A., Migliore E., Carosso A., Piccioni P., Cavallero M., Caria E., Salamina G., Arossa W. (2003). Tuberculin reactivity in adult BCG-vaccinated subjects: A cross sectional study. Int. J. Tuberc. Lung Dis..

[31] Bierrenbach A.L., Floyd S., Cunha S.C., Dourado I., Barreto M.L., Pereira S.M., Hijjar M.A., Rodrigues L.C. (2003). A comparison of dual skin test with mycobacterial antigens and tuberculin skin test alone in estimating prevalence of *Mycobacterium tuberculosis* infection in population surveys. Int. J. Tuberc. Lung Dis..

[32] Rangaka M.X., Wilkinson K.A., Glynn J.R., Ling D., Menzies D., Mwansa-Kambafwile J., Wilkinson R.J., Pai M. (2012). Predictive value of interferon-γ release assays for incident active tuberculosis: A systematic review and meta-analysis. Lancet Infect. Dis..

[33] Zwerling A., van den Hof S., Scholten J., Cobelens F., Menzies D., Pai M. (2012). Interferon-gamma release assays for tuberculosis screening of healthcare workers: A systematic review. Thorax.

[34] Rangaka M.X., Wilkinson K.A., Seldon R., Van Cutsem G., Meintjes G.A., Morroni C., Mouton P., Diwakar L., Connell T.G., Maartens G. (2007). Effect of HIV-1 infection on T-Cell-based and skin test detection of tuberculosis infection. Am. J. Respir. Crit. Care Med..

[35] Dorman S.E., Belknap R., Graviss E.A., Reves R., Schluger N., Weinfurter P., Wang Y., Cronin W., Hirsch-Moverman Y., Teeter L.D. (2014). Interferon-gamma release assays and tuberculin skin testing for diagnosis of latent tuberculosis infection in healthcare workers in the United States. Am. J. Respir. Crit. Care Med..

[36] Zwerling A., Benedetti A., Cojocariu M., McIntosh F., Pietrangelo F., Behr M.A., Schwartzman K., Menzies D., Pai M. (2013). Repeat IGRA testing in Canadian health workers: Conversions or unexplained variability?. PLoS ONE.

[37] Slater M.L., Welland G., Pai M., Parsonnet J., Banaei N. (2013). Challenges with QuantiFERON-TB Gold Assay for Large-Scale, Routine Screening of US Healthcare Workers. Am. J. Respir. Crit. Care Med..

[38] Fong K.S., Tomford J.W., Teixeira L., Fraser T.G., van Duin D., Yen-Lieberman B., Gordon S.M., Miranda C. (2012). Challenges of interferon-gamma release assay conversions in serial testing of health-care workers in a TB control program. Chest.

[39] Tudor C., van der Walt M.L., Margot B., Dorman S.E., Pan W.K., Yenokyan G., Farley J.E. (2016). Occupational Risk Factors for Tuberculosis Among HCWs. Clin. Infect. Dis..

[40] Adams S., Ehrlich R., Baatjies R., Dendukuri N., Wang Z., Dheda K. (2019). Predictors of discordant latent tuberculosis infection test results amongst South African health care workers. BMC Infect. Dis..

[41] Churchyard G.J., Chaisson R.E., Maartens G., Getahun H. (2014). Tuberculosis preventive therapy: An underutilized strategy to reduce individual risk of TB and contribute to TB control. S. Afr. Med. J..

[42] Churchyard G.J., Fielding K.L., Grant A.D. (2014). A trial of mass isoniazid preventive therapy for tuberculosis control. N. Engl. J. Med..

